# A latent profile analysis of sleep disturbance in relation to mental health among college students in China

**DOI:** 10.3389/fpubh.2023.1107692

**Published:** 2023-05-31

**Authors:** Chunping Chen, Zigeng He, Bingna Xu, Jianyao Shao, Dongfang Wang

**Affiliations:** ^1^Institute of Education, Xiamen University, Xiamen, China; ^2^School of Psychology, Centre for Studies of Psychological Applications, Guangdong Key Laboratory of Mental Health and Cognitive Science, Ministry of Education Key Laboratory of Brain Cognition and Educational Science, South China Normal University, Guangzhou, China

**Keywords:** sleep disturbance, depressive symptoms, psychotic-like experience, resilience, college students

## Abstract

**Aims:**

This study aimed to examine the subtype classification characteristics of sleep disturbance (SD) in college students and their associations with sample characteristic factors and mental health outcomes.

**Methods:**

The sample comprised 4,302 college students (Mean age = 19.92 ± 1.42 years, 58.6% females). The Youth Self-Rating Insomnia Scale, Beck Depression Inventory, 8-item Positive Subscale of the Community Assessment of Psychic Experiences, and 10-item Connor-Davidson Resilience Scale were used to assess adolescents’ sleep disturbance, depressive symptoms, psychotic-like experiences (PLEs), and resilience. Latent profile analysis, logistic regression, and liner regression analysis were used to analyze the data.

**Results:**

Three subtypes of SD in college students were identified: the high SD profile (10.6%), the mild SD profile (37.5%), and the no SD profile (51.9%). Compared with college students in the “no SD” profile, risk factors for “high SD” include being male and poor parental marital status. Sophomores were found to predict the “high SD” profile or “mild SD” profile relative to the “no SD” profile. College students in the “mild SD” profile or “high SD” profile were more likely to have a higher level of depressive symptoms and PLEs, while a lower level of resilience.

**Conclusion:**

The findings highlighted that target intervention is urgently needed for male college students, sophomores, and those with poor parental marital status in the “mild SD” profile or “high SD” profile.

## Introduction

Sleep disturbance (SD) is prevalent among adolescents and young adults, with main symptoms of insomnia, poor sleep quality, and other sleep complaints. College students, one of the most sleep deprived student groups, are particularly vulnerable to disturbed sleep. One epidemiological survey showed that more than 60% of college students were suffering from SD [on the Pittsburgh Sleep Quality Index (PSQI)] (1). Gaultney, in a sample of 1845 college students, found that 27% have clinically significant symptoms of SD (on the SLEEP-50 questionnaire) (2). Recent systematic review, involving 250 studies, estimated that global prevalence of SD among college students was 41.16% during the COVID-19 pandemic (3). Adolescents entering college is a major life transition, which means leaving home and living independently, completing demanding course and trying to earn academic degrees, facing a new social environment, and adopting new social roles (4–6). This transition contributes to increased stress levels, which, in turn, can have a profound and lasting effect on sleep (7). Moreover, studies have also shown that excessive media use by college students contributes to poor sleep hygiene and a series of sleep disturbances (8).

A growing number of research have focused on SD of college students and its symptoms, such as insomnia (difficulty initiating sleep, difficulty maintaining sleep, or early morning awakening) (9), poor subjective sleep quality (10, 11), daytime sleepiness (12), salient nightmares (13), and other general sleep problems (14). The majority of extant research on SD has relied on variable-centered analyses, which provides insight into the average relations among symptoms or diagnoses of SD within a specific group. However, the methodological approaches fail to reveal different patterns between individuals and may therefore draw over-generalized conclusions based on overall samples (15). On the contrary, the use of person-centered approaches such as latent class analysis (LCA) and latent profile analysis (LPA), to evaluate the structure of psychopathology, enables a more refined understanding of symptom presentations and is ideal for psychopathology research (16, 17).

Till now, the heterogeneity of SD for college students has only been scarcely explored. Based on the available literature, only three studies have been conducted using a person-centered methodological approach to explore the heterogeneous patterns of sleep-related impairment among college students. For instance, Carpi et al. (18) identified a five-class solution among a small sample of college students (N = 490) by using LPA, which were termed as the “severe insomnia” subtype, “moderate insomnia with medication use” subtype, “subthreshold insomnia” subtype, “subthreshold insomnia with sleep latency complaints” subtype, and “moderate insomnia with sleep duration complaints” subtype (18). Zhou et al. (19) applied LCA to discern heterogeneous patterns of sleep behaviors among 1,288 Chinese college students, and summarized four subtypes: good sleep, prolonged sleep latency, sleep disturbances–daytime dysfunction, and multiple poor sleep behavior (19). Another study in 312 at-risk college drinkers also indicted that the four profiles can clearly explain sleep and sleep-related consequences (20). Therefore, further studies with a large sample are needed to confirm heterogeneous patterns of SD among general college students.

This study intended to explore the heterogeneity of SD among the college students group with a large sample web-based survey for college students. As suggested by prior research, the risks of occurrence and persistence of SD are associated with numerous sample characteristic factors, including age (21, 22), sex (23, 24), parental marital status (25), and family economic status (26). Additionally, solid evidence showed that SD is also associated with a wide variety of adverse mental health outcomes, such as depression (27) and psychotic-like experiences (PLEs) (28), as well as inhibits the development of positive mental qualities, such as resilience (29). Collectively, there are three main aims of this study. First, the study aims to identify the heterogeneity differences in SD among college students, employing LPA. Second, it explores what sample characteristic factors (e.g., sex, age) are significant predictors of distinct profiles of SD in college students. Finally, it examines the associations among these different subtypes of SD and mental health outcomes. Based on previous research, we speculated that the college students’ SD feature obvious latent profiles, and several sample characteristic factors, such as sex, parental marital status, were significant predictors of distinct profiles for SD. We also anticipated that there are robust associations between SD subtypes and depression, PLEs and resilience.

## Materials and methods

### Participants and procedure

A convenience sample of college students was recruited from Taiyuan city in Shanxi Province, China. The online survey was conducted during January 2021, using the “Questionnaire Star” software platform. All participants scanned the Quick Response (QR) code of the questionnaire through their mobile phones to complete the web-based survey. If respondents agree to participate in this study, they shall select the “I agree to participate” option and upload their electronic signature of informed consent on the first page of the questionnaire platform. The investigation used the principle of voluntary participation, and participants could withdraw from the survey at any time if they felt discomfort. To improve data quality, inclusion criteria for participation included: (a) upload the electronic signed version of the informed consent form; (b) response time for web-based survey was above 5 min; and (c) have no current significant physical disease or history of psychiatric illness. Finally, 4,302 responses were qualified and included in the subsequent analyses.

The current study was carried out in accordance with the Helsinki Declaration as revised in 1989 and was approved by the Ethics Committees of South China Normal University.

### Measures

#### SD

The Chinese version of Youth Self-Rating Insomnia Scale (YSIS) was used to measure the SD in the past month (30). It consists of 8 items, clustering into two dimensions, namely daytime distress/impairment and insomnia symptoms. Each item scored from 1 to 5, and a higher total score indicate a greater level of SD. The cut-off point of 26 represents identified cases of clinically SD (30). In this study, Cronbach’s α was 0.89.

#### Depressive symptoms

The Beck Depression Inventory (BDI) was used to assess depressive symptoms in the past week (31). It consists of 21 items, and each item was rated on a four-point Likert scale, from 0 to 3, and a higher total score suggested a greater level of depressive symptoms. A cut-off score of 14 was suggested to identify the clinically significant depression (32).The Chinese version of BDI-21 has demonstrated good internal reliability and the concurrent validity (33), and Cronbach’s α was 0.91 in this sample.

#### PLEs

The Chinese version of 8-item positive subscale of the community assessment of psychic experiences (CAPE-P8) was used to evaluate frequency of PLEs in the past month (34, 35). CAPE-P8 originated from 42-item community assessment of psychic experiences (36, 37), which addresses the following domains, namely delusional experiences and hallucinatory experiences. Responses to items range from 1—never, 2—sometimes, 3—often, to 4—nearly always, with a higher score reflecting more frequent PLEs. Frequent PLEs were defined as having “often” or “nearly always” on one or more items (38, 39), and Cronbach’s α was 0.84 in the current sample.

#### Resilience

The 10-item Connor-Davidson Resilience Scale (CD-RISC-10) was used to assess the level of resilience (40). Five response options are available on a scale of 0 (not true at all) to 4 (true nearly all the time). Higher score means higher level of resilience. Psychometric properties of the CD-RISC-10 have been described in the Chinese population (41, 42). In our study, Cronbach’s α was rather high (α = 0.97).

#### Sample characteristic variables

Sample characteristic variables were collected in a self-report fashion, including sex [1 = male; 2 = female], age, grade [1 = freshman; 2 = sophomore; 3 = junior; 4 = senior], ethnicity [1 = Han (the major ethnic group in China); 2 = others], parental married status [1 = good; 2 = poor (separated, divorced, and widowed)], family income [1 = <3,000 RMB; 2 = 3,000 ~ 5,000 RMB; 3= >5,000 RMB], parents’ education level [1 = junior high school or less; 2 = senior high school; 3 = college or more], and single child status [1 = yes; 2 = no].

### Statistical analysis

Data analysis was performed using SPSS 24.0 and Mplus 8.30. First, the Harman’s one-factor test through exploratory factor analysis (EFA) was performed to examine common method bias (43). The EFA found out that there were 5 factors with eigenvalues >1 and the first factor accounted for 11.17% of the total variance, indicating common method bias was not a serious issue in the study (44). Second, the LPA was conducted to determine whether there were heterogeneous latent classification differences in SD among college students. LPA is a form of LCA that is used when working with continuous variables. The fit of a one-class model is initially evaluated, and models with increasing number of latent profiles were estimated. To identify the optimal number of latent profiles to fit the data, we compared several fit indicates of each model, including Akaike’s information criterion (AIC), the Bayesian information criterion (BIC), sample-size-adjusted Bayesian information criterion (aBIC), Entropy, Vuong-Lo–Mendell–Rubin Likelihood Ratio Test (VLMR-LRT), and bootstrapped likelihood ratio test (BLRT). Lower AIC, BIC, and aBIC values (45), higher entropy values (46), and significant VLMR-LRT and BLRT *p*-values (47) were considered as signs of a better fit. In addition, we took interpretability of the profiles into great consideration, and ensured that each profile consisted of no less than 5% of samples (48). Third, multivariate logistic regression analyses were implemented to test whether sample characteristic variables differentially predicted SD profile membership. Finally, YSIS, BDI-21, CAPE-P8, and CD-RISC-10 total scores were compared through the ANOVA test. We also treated the subtypes of SD as dummy variables and further examined the relationship between SD subtypes and depression, PLEs and resilience using linear regression. A two-tailed *p* < 0.05 was considered statistically significant.

## Results

### Description of the sample

In total, 4,302 participants completed questionnaires, among which 2,522 were female. The mean age of the sample was 19.92 ± 1.42 years. A majority of college students was ethnicity Han (99.4%), and reported with a good parental marital status (93.1%). Other sample characteristic variables are presented in Table 1.

**Table 1:** Sample characteristics (*N* = 4,302).

Characteristics		*N*	%
Sex	Male	1780	41.4
	Female	2,522	58.6
Age [year, Mean (SD)]	19.92(1.42)		
Grade	Freshman	1876	43.6
	Sophomore	1,133	26.3
	Junior	1,044	24.3
	Senior	249	5.8
Ethnicity	Han ^a^	4,277	99.4
	Others	25	0.6
Parental marital status	Good	4,005	93.1
	Poor ^b^	297	6.9
Family income (monthly), RMB	<3,000	2,419	56.2
	3,000 ~ 5,000	1,153	26.8
	>5,000	730	17.0
Father’s education	Junior high school or less	2,831	65.8
	Senior high school	914	21.2
	College or more	557	12.9
Mother’s education	Junior high school or less	3,009	69.9
	Senior high school	846	19.7
	College or more	447	10.4
Single child status	Yes	1,107	25.7
	No	3,195	74.3
YSIS score[Mean (SD)]	14.53(5.77)		
Sleep disturbance ^c^	Yes	196	4.6
BDI score [Mean (SD)]	3.45(5.76)		
Depression^d^	Yes	293	6.8
CAPE-P8 score[Mean (SD)]	9.20(2.20)		
Psychotic-like experiences^e^	Yes	209	4.9
Resilience [Mean (SD)]	30.66(7.92)		

YSIS, Youth Self-Rating Insomnia Scale; BDI, Beck Depression Inventory; CAPE-P8, 8-item Positive Subscale of the Community Assessment of Psychic Experiences; CD-RISC-10, 10-item Connor-Davidson Resilience Scale.

a Han is the major ethnic group in China.

b Poor parental marital status included separated, divorced, and widowed.

c Sleep disturbance calculated using the YSIS, with a clinical cut-off score of 26.

d Depression calculated using the BDI-21, with a clinical cut-off score of 14.

e Psychotic-like experiences calculated using the CAPE-P8, with one or more items were selected 3-often or 4-nearly always.

Table 1 also displays 4.6% of participants (*N* = 196) reported the presence of clinically SD. 6.8% (*N* = 293) had depression, and 4.9% (*N* = 209) respondents were identified as frequents PLEs.

### Model fitting

Based on the 8 items of the YSIS, a potential profile analysis was conducted, which, in turn, established four latent profile models. The results of the LPA are reported in Table 2. The VLMR-LRT and BLRT values were statistically significant for two and three-category model. Moreover, the fit of the three-category model was better than a two-category model, as indicated by the lower AIC and BIC values. Also, Entropy value for three-category model was 0.89, indicating a high accuracy of these classifications. Therefore, the three-category model was selected as the optimal model. The average probability of samples belonging to each potential category was between 92.2 and 96.8%.

**Table 2:** Fit indices for latent profile analyses.

Class	BIC	aBIC	AIC	BLRT (*p*)	Entropy	aLMR (*p*)	VLMR (*p*)	Smallest class (%)
1	93415.61	93364.77	93313.74					
2	81361.89	81282.45	81202.71	<0.001	0.89	<0.001	<0.001	33.3
**3**	**77743.38**	**77635.34**	**77526.91**	**<0.001**	**0.89**	**<0.001**	**<0.001**	**10.6**
4	75615.38	75478.74	75341.61	<0.001	0.96	0.257	0.254	9.9

Bold indicates best fit. Entropy and value of *p* for BLRT, aLMR, and VLMR are not applicable to a one-class solution. BIC, Bayesian information criterion; aBIC, sample-size-adjusted Bayesian information criterion; AIC, Akaike information criterion; BLRT, bootstrap likelihood ratio test; aLMR, adjusted Lo–Mendell–Rubin; VLMR, Vuong-Lo–Mendell–Rubin.

### Sleep disturbance profiles characterization

**Figure 1:**
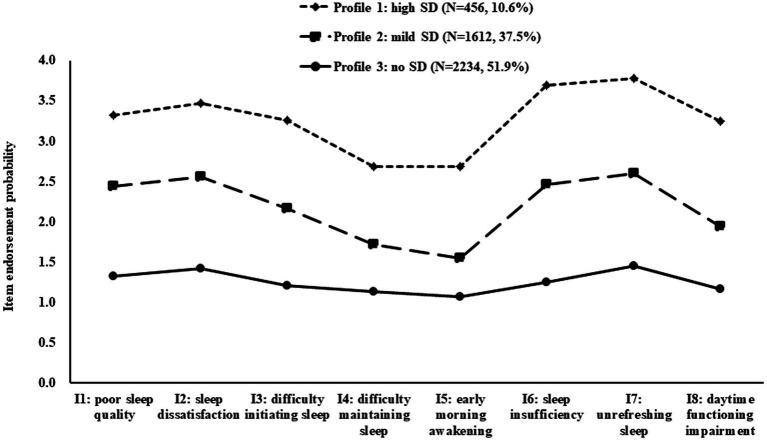
Mean sleep disturbance symptom cluster scores in the three latent profiles. SD, sleep disturbance.

A latent class profile plot of the sample is shown in Figure 1. Profile 1 labeled as the “high SD” profile (N = 456, accounting for 10.6% of the total sample) was characterized by the highest level of SD, and its YSIS total score (26.12 ± 3.53) > 26 (the cut-off point). Profile 3 defined as the “no SD” profile (N = 2,234, 51.9%) was characterized by the lowest level of SD, and its YSIS total score (10.05 ± 1.95) much lower than the sample mean value (14.53 ± 5.77). Additionally, profile 2 comprised approximately 37.5% of the sample (N = 1,612) and included college students with a higher level of SD compared to the sample reported in profile 3, while lower than those belonging to profile 1. The YSIS total score of this profile (17.45 ± 2.37) slightly above the sample mean value (14.53 ± 5.77). This profile was named as “mild SD” profile.

### Profile predictors and outcomes

Taking the profile of SD as the dependent variable, the “no SD” profile was used as the reference category. Sex, age, grade, ethnicity, parental married status, family income, parents’ education level, and single child status were used as independent variables to carry out a multivariate logistic regression. The results (Table 3) showed that, compared with college students in the “no SD” profile, risk factors for “high SD” include being male (OR = 1.24, 95% CI = 1.01–1.53) and poor parental marital status (OR = 1.81, 95% CI = 1.27–2.59). Meanwhile, sophomores tended to predict the “high SD” profile (OR = 1.44, 95% CI = 1.11–1.88) or “mild SD” profile (OR = 1.22, 95% CI = 1.03–1.45) relative to the “no SD” profile.

**Table 3:** Predictors associated with profile membership of SD [OR(95%CI)].

Characteristics		Profile 1 high SD	Profile 2 mild SD
Sex	Female	Ref.	Ref.
	Male	1.24 (1.01, 1.53)^*^	0.93(0.82, 1.06)
Age		0.95(0.86,1.06)	0.98(0.92,1.05)
Grade	Freshman	Ref.	Ref.
	Sophomore	1.44 (1.11, 1.88)^**^	1.22 (1.03, 1.45)^*^
	Junior	0.92(0.65,1.31)	0.95(0.77,1.18)
	Senior	0.75(0.41,1.37)	0.73(0.50,1.05)
Ethnicity	Han	Ref.	Ref.
	Others	0.67 (0.15,2.98)	0.91 (0.39,2.12)
Parental marital status	Good	Ref.	Ref.
	Poor	1.81 (1.27, 2.59)^***^	1.29 (1.00, 1.67)
Family income (monthly), RMB	<3,000	Ref.	Ref.
	3,000 ~ 5,000	0.87 (0.68,1.12)	0.82 (0.82, 1.11)
	>5,000	1.17 (0.88,1.56)	1.01 (0.84, 1.23)
Father’s education	Junior high school or less	Ref.	Ref.
	Senior high school	0.99 (0.75, 1.30)	0.99 (0.83, 1.17)
	College or more	0.96 (0.66, 1.39)	0.88 (0.69, 1.13)
Mother’s education	Junior high school or less	Ref.	Ref.
	Senior high school	0.96 (0.72, 1.28)	0.88 (0.73, 1.06)
	College or more	1.08 (0.72, 1.60)	0.59 (0.59, 1.01)
Single child status	Yes	Ref.	Ref.
	No	0.86 (0.68, 1.10)	0.93 (0.80, 1.09)

The reference category for the dependent variables was the “Profile 3: no SD”; SD = sleep disturbance; OR = odds ratio; CI = confidence interval; ^*^p < 0.05, ^**^p < 0.01, ^***^p < 0.001; Bold type indicates a significant odds ratio.

**Figure 2:**
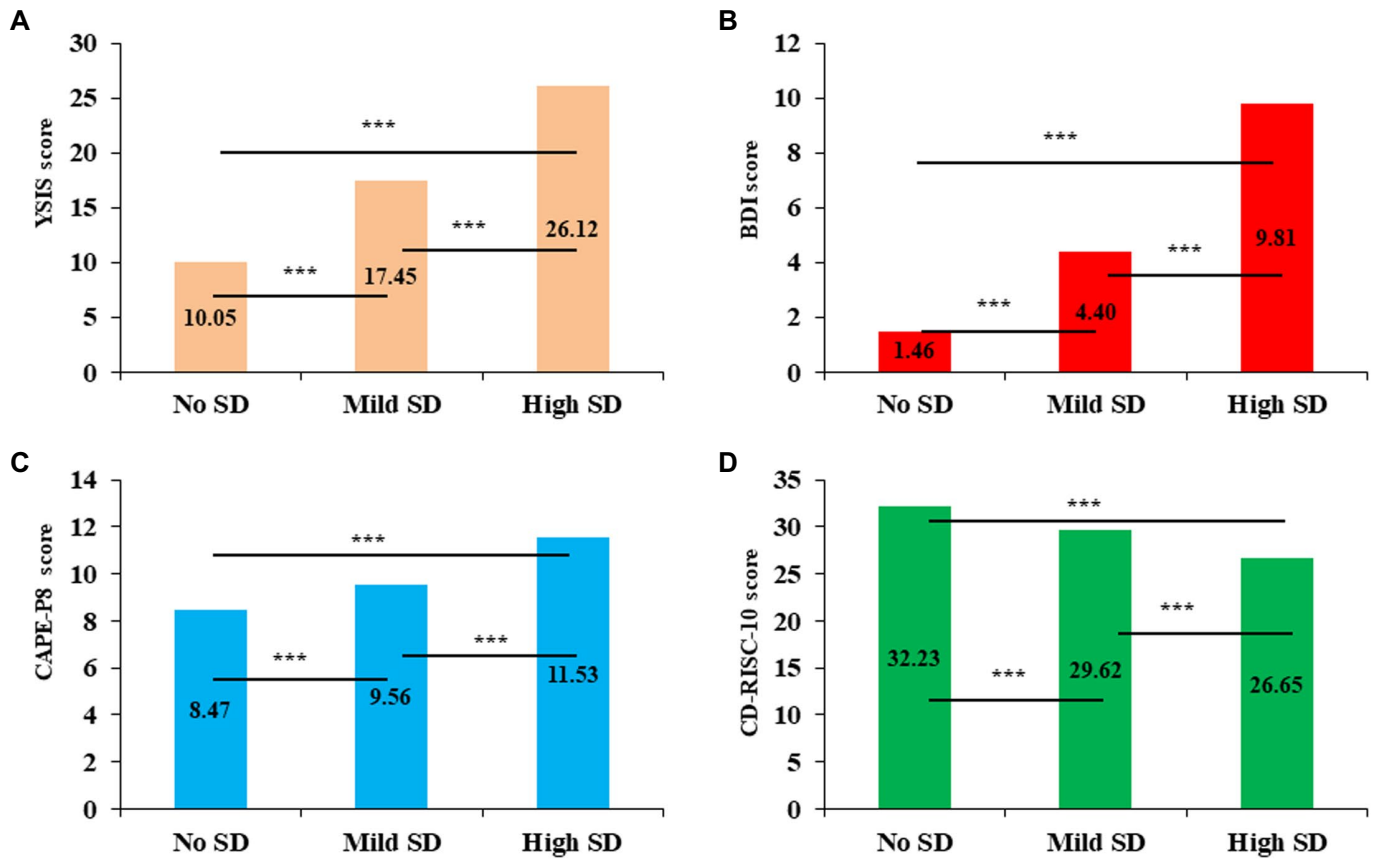
Comparison of mental health variables across different SD profiles. SD, sleep disturbance; YSIS, Youth Self-Rating Insomnia Scale; BDI, Beck Depression Inventory; CAPE-P8, 8-item Positive Subscale of the Community Assessment of Psychic Experiences; CD-RISC-10, 10-item Connor-Davidson Resilience Scale. ^***^p < 0.001.

Figure 2 compares YSIS, BDI-21, CAPE-P8, and CD-RISC-10 total scores among the three profile of SD. The “high SD” profile had higher YSIS (*F* = 11119.46, *p* < 0.001, Figure 2A), BDI-21 (*F* = 541.26, *p* < 0.001, Figure 2B), and CAPE-P8 scores (*F* = 491.81, *p* < 0.001, Figure 2C) while lower CD-RISC-10 score (*F* = 123.34, *p* < 0.001, Figure 2D) than the other two profiles. The relations among profiles and mental health outcomes are described in Table 4. In the crude model, college students in the “mild SD” profile or “high SD” profile were more likely to have a higher level of depressive symptoms and PLEs, while a lower level of resilience compared with those in the “no SD” profile. This significant effect between the mild/high SD and college students’ mental health persisted after adjusting for sample characteristics variable.

**Table 4:** Sleep disturbance profiles in relation to mental health.

		Depressive symptoms			PLEs			Resilience		
	Predictor	*β*	95%CI	*p*	*β*	95%CI	*p*	*β*	95%CI	*p*
Crude model	Profile 3: no SD	Ref.			Ref.			Ref.		
	Profile 2: mild SD	0.25	1.25,1.68	<0.001	0.24	0.96,1.21	<0.001	−0.16	−3.11,-2.13	<0.001
	Profile 1: high SD	0.45	7.82,8.86	<0.001	0.43	2.86,3.26	<0.001	−0.22	−6.36,-4.81	<0.001
Adjusted model^a^	Profile 3: no SD	Ref.			Ref.			Ref.		
	Profile 2: mild SD	0.25	2.58,3.24	<0.001	0.24	0.95,1.20	<0.001	−0.16	−3.06,-2.07	<0.001
	Profile 1: high SD	0.44	7.71,8.74	<0.001	0.42	2.82,3.22	<0.001	−0.22	−6.32,-4.76	<0.001

a Adjusted for sample characteristics in Table 1.

CI, confidence interval; PLEs, Psychotic-like experiences; SD, sleep disturbance.

## Discussion

The present study adopted LPA to explore subtypes of SD and their related factors in college students. The findings are beneficial to education administrators and clinicians better understand the heterogeneous cluster classification characteristics of SD in college students to formulate more specific and targeted intervention measures.

First, three latent profiles of SD among college students were identified in this study: “high SD,” “mild SD,” and “no SD.” The finding of the presence of three potential SD profiles is similar to previous studies in other groups (49, 50). For example, Tejada and colleagues explored the heterogeneity of SD in 1,331 chemotherapy patients using LPA, pointing out three SD profiles: “low,” “high,” and “very high” (50). Our data showed that approximately half of the samples (51.9%) were categorized as “no SD” profile, and only 10.6% have high SD. We compared the results with previous studies of similar university students. Zhou and colleagues used LCA to analyze the distinct classes of sleep behavior (on the Dysfunction Beliefs and Attitudes about Sleep [DBAS] and PSQI), and concluded that 6.8% of college students was in the “sleep disturbances and daytime dysfunction” subtype, while 31.8% was in the “good sleep” subtype (19). Another study (on the Insomnia Severity Index [ISI] and PSQI) adapted LPA and pointed out that 8.8% college students have severe insomnia, 66.9% reported subthreshold insomnia or subthreshold insomnia with sleep latency complaints (composed by students with relatively low insomnia severity) (18). Due to instruments and sampling, the findings differ to some extent. However, these studies have reached consistent conclusions, that is, most individuals could cope well to stressor of life events and maintain good sleep function, while only a small percentage exhibit clinical levels of SD. In addition, 37.5% of the samples were categorized as “mild SD” profile. Although the YSIS scores of these college students did not meet the criteria for clinically SD (cut-off value = 26), they had higher than average levels of SD. College students in “mild SD” profile still exhibited low frequency (the average score of the 8 YSIS item was 2.18) of SD related symptoms. Therefore, it is necessary to employ targeted interventions for those in this subtype to prevent them from sliding into sleep-related impairment and SD.

Second, multivariate logistic regression identified several risk factors associated with increased likelihood of experiencing moderate and high SD. Our data showed that male students were more likely to be found in the “high SD” profile than the female. Although previous studies have examined SD in the college students group, findings concerning the effects of sex on SD have been inconsistent. Several studies found that male students exhibiting greater SD (51–53). However, some studies suggested that SD among female students was even more severe than that of the male (23, 54, 55), while others reported no sex differences in SD (56–58). We speculated that male college students have more sleep-adverse lifestyles, such as Internet addiction, alcohol and energy drinks consumption, and cigarette smoking (59, 60). Poor parental marital status was also one of the risk factors for SD, in line with previous research (25, 61). Stressful changes in the parents’ relationship lead to disruptions in family processes, which, in turn, negatively impact the individual’s physiologic functioning (62, 63), and physiologic changes may interfere with sleep (64, 65). The results also indicate the possible impacts of the sophomores on the development of SD among college students. The finding is similar with previous study (66), in which sophomores had more professional course loads compared with freshmen, and these stress may have affected their sleep quality to some extent.

Third, college students in the “mild SD” and “high SD” profile report greater depressive symptoms and PLEs in comparison to those in the no SD profile. A great volume of literature has consistently asserted that SD as a risk factor for depressive symptoms and PLEs (28, 67, 68). Recent evidence has also suggested that college students with SD are prone to maladaptive emotion regulation (69) and impaired social functioning (70), resulting in the development of depressive symptoms and PLEs. Additionally, our study also found SD negatively associated with resilience. Studies in children have reported that greater SD reduced resilience (71). Another longitudinal study also found that higher sleep rhythmicity and fewer SD in early age predicted higher behavioral control in adolescence, which, in turn, predicted resilience in young adulthood (72).

Accordingly, therapeutic interventions targeting SD may benefit from a tailored approach that takes individual symptom patterns of SD into great consideration. For example, college students in profile 1 (high SD) have poor sleep quality and more adverse psychological outcomes, therefore require prompt clinical treatment to help alleviate their sleep disorders. Meanwhile, although college students in profile 1 (mild SD), who did not have particularly severe SD related symptoms, were still at higher risk for mental health problems relative to those in the no SD profile. Therefore, it is necessary to optimize the sleep habits of these students and improve their sleep quality. Several interventions can be carried out (e.g., cognitive behavioral therapy, mindfulness and hypnotherapy (73)) to improve sleep quality for college students in the mild SD and high SD profile. Also, influential factors should also be taken into consideration for effective psychosocial intervention for college students. Some students, especially those with male gender, sophomores and poor parental marital status, exhibit moderate or high SD. Thus, the need for individualized intervention with these students is indicated.

## Limitations

Despite all the relevant findings, several limitations of the current study should be noted. First, measures of SD, depressive symptom, PLEs, and other psychological factors relied on self-report questionnaires, which might be influenced by reporting bias caused by recollection inaccuracy and individuals’ own psychiatric states. Second, the data were collected in the Shanxi Province of China, which is uncertain whether the findings could be generalized to all college students to other regions of China. Thus, future studies would benefit from increasing the sampling range and sample size. Finally, this was a cross-sectional study, which limits the ability to make causal inference. In clinical practice, SD and mental health may do form a bidirectional relationships (74–76). Therefore, future research is necessary to design longitudinal studies to further explore the effects of different SD subtypes on mental health outcomes.

## Conclusion

These findings indicate that SD in Chinese college students has obvious classification characteristics. We identified three profiles of SD in college students. Furthermore, we suggest that education or clinical workers should pay particular attention to college students classified into the mild SD and high SD profile and especially male college students, sophomores and those with poor parental marital status.

## Data availability statement

The original contributions presented in the study are included in the article/supplementary material, further inquiries can be directed to the corresponding author.

## Ethics statement

The studies involving human participants were reviewed and approved by Ethics Committees of South China Normal University. The patients/participants provided their written informed consent to participate in this study.

## Author contributions

DW: conceptualization. CC and DW: methodology, formal analysis, and writing—original draft. ZH, BX, and JS: data curation. BX and DW: writing–review and editing. All authors contributed to the article and approved the submitted version.

## Funding

The present study was funded by Graduate Research and Innovation Project of School of Psychology, South China Normal University (PSY-SCNU202017).

## Conflict of interest

The authors declare that the research was conducted in the absence of any commercial or financial relationships that could be construed as a potential conflict of interest.

## Publisher’s note

All claims expressed in this article are solely those of the authors and do not necessarily represent those of their affiliated organizations, or those of the publisher, the editors and the reviewers. Any product that may be evaluated in this article, or claim that may be made by its manufacturer, is not guaranteed or endorsed by the publisher.
